# The PRR14 heterochromatin tether encodes modular domains that mediate and regulate nuclear lamina targeting

**DOI:** 10.1242/jcs.240416

**Published:** 2020-05-27

**Authors:** Kelly L. Dunlevy, Valentina Medvedeva, Jade E. Wilson, Mohammed Hoque, Trinity Pellegrin, Adam Maynard, Madison M. Kremp, Jason S. Wasserman, Andrey Poleshko, Richard A. Katz

**Affiliations:** 1 Cancer Epigenetics Program, Fox Chase Cancer Center, Temple University Health System, Philadelphia, PA 19111, USA; 2 Department of Cell and Developmental Biology, Perelman School of Medicine, University of Pennsylvania, Philadelphia, PA 19104, USA

**Keywords:** Chromatin organization, Heterochromatin, Nuclear lamina, PRR14, Lamina binding domain

## Abstract

A large fraction of epigenetically silent heterochromatin is anchored to the nuclear periphery via ‘tethering proteins’ that function to bridge heterochromatin and the nuclear membrane or nuclear lamina. We previously identified a human tethering protein, PRR14, that binds heterochromatin through an N-terminal domain, but the mechanism and regulation of nuclear lamina association remained to be investigated. Here we identify an evolutionarily conserved PRR14 nuclear lamina binding domain (LBD) that is both necessary and sufficient for positioning of PRR14 at the nuclear lamina. We show that PRR14 associates dynamically with the nuclear lamina, and provide evidence that such dynamics are regulated through phosphorylation and dephosphorylation of the LBD. Furthermore, we identify a PP2A phosphatase recognition motif within the evolutionarily conserved C-terminal Tantalus domain of PRR14. Disruption of this motif affects PRR14 localization to the nuclear lamina. The overall findings demonstrate a heterochromatin anchoring mechanism whereby the PRR14 tether simultaneously binds heterochromatin and the nuclear lamina through two separable modular domains. Our findings also describe an optimal PRR14 LBD fragment that could be used for efficient targeting of fusion proteins to the nuclear lamina.

## Abstract (precis)

**Summary:** We have identified an evolutionarily conserved nuclear lamina binding domain in the PRR14 nuclear lamina–heterochromatin tethering protein.

## INTRODUCTION

The eukaryotic nuclear genome is organized into chromatin and is enclosed by the nuclear envelope that forms the border of the nuclear organelle. The nuclear envelope is a double membrane (Buchwalter et al., 2019; Hetzer, 2010; Ungricht and Kutay, 2015) that, in multicellular organisms, includes the nuclear lamina protein framework that lies underneath the inner nuclear membrane (Dittmer and Misteli, 2011; Gruenbaum and Foisner, 2015). The nuclear lamina is composed of the intermediate filament proteins Lamin A, Lamin C, Lamin B1 and Lamin B2. In addition to acting as a nuclear framework, the nuclear lamina serves as a docking site for heterochromatin, which forms a characteristic silent ‘peripheral heterochromatin compartment’ decorated with the repressive histone tail modifications histone 3 lysine 9 di- and tri-methylation (H3K9me2 and H3K9me3) (Buchwalter et al., 2019; Gordon et al., 2015; Gruenbaum and Foisner, 2015; Lemaître and Bickmore, 2015; Meister and Taddei, 2013; Padeken and Heun, 2014; Poleshko et al., 2019; Politz et al., 2013; Shevelyov and Nurminsky, 2012; Shevelyov and Ulianov, 2019; van Steensel and Belmont, 2017; Wong and Reddy, 2015). This heterochromatin compartment, positioned at the nuclear lamina, is the focus of much interest as it provides a critical function in housing lineage-inappropriate repressed genes and gene-poor DNA (Becker et al., 2016; Peric-Hupkes et al., 2010; Poleshko et al., 2017; Reddy et al., 2008; Stancheva and Schirmer, 2014; Zhou et al., 2011). Furthermore, perturbations of heterochromatin or gene positioning at the nuclear periphery may contribute to disease and aging (Bank and Gruenbaum, 2011; Butin-Israeli et al., 2012; Davidson and Lammerding, 2014; Dittmer and Misteli, 2011). Although there has been significant progress in mapping local and global 3D chromatin organization (Dekker et al., 2017), an understanding of the mechanisms underlying such organization is limited. Regarding peripheral heterochromatin organization, historical and emerging findings, including our own, have shown that proteins function as ‘tethers’ to attach heterochromatin to the nuclear periphery.

Early studies identified the Lamin B Receptor (LBR) (Olins et al., 2010; Ye and Worman, 1996) as a multi-pass inner nuclear membrane protein that binds to peripheral H3K9me3 heterochromatin through the prolific adapter protein, heterochromatin protein 1 (HP1) (Canzio et al., 2014; Lomberk et al., 2006; Zeng et al., 2010). The HP1 bivalent adapter function is mediated by an N-terminal chromodomain (CD) that is a ligand for H3K9me2/3 and a C-terminal chromoshadow domain that recruits numerous PxVxL motif-containing partners to heterochromatin (Lechner et al., 2005; Machida et al., 2018; Nozawa et al., 2010). Using a cell-based epigenetic silencing factor screen, we previously identified an unstudied, widely expressed, 585-amino-acid human protein, Proline-rich protein 14 (PRR14) (Poleshko and Katz, 2014; Poleshko et al., 2014, 2013). Using confocal imaging, PRR14 was found to localize strongly to the inner nuclear periphery. Earlier, PRR14 had been detected as a binding partner of HP1 in two independent screens (Nozawa et al., 2010; Rual et al., 2005). We identified a modular N-terminal heterochromatin-binding domain of PRR14 (amino acids 1–135) that contains a functional HP1-partner LAVVL motif at positions 52–56. This LAVVL motif fits well among variants of the classic PxVxL motif (Lechner et al., 2005; Machida et al., 2018; Nozawa et al., 2010; Romeo et al., 2015). We hypothesized that PRR14 could thereby function as a tether to position HP1-bound H3K9me3 heterochromatin at the nuclear lamina, and PRR14 knockdown experiments demonstrated such a role (Poleshko et al., 2013).

LBR and PRR14 proteins are now established as functioning to tether heterochromatin at the nuclear periphery (Shevelyov and Ulianov, 2019). The tethering mechanism implicates bivalent attachment to the nuclear periphery and heterochromatin. During interphase, the LBR multi-pass integral membrane protein is anchored to the inner nuclear membrane (Olins et al., 2010). Unlike LBR, which remains membrane-associated during mitosis, PRR14 was found to be soluble in metaphase and to colocalize with HP1 on chromosomes immediately at the onset of anaphase (Poleshko et al., 2013). Importantly, we found that during interphase, PRR14 localization at the nuclear periphery is disrupted by siRNA knockdown of the nuclear lamina components Lamin A and Lamin C, but not by knockdown of Lamin B1 or Lamin B2 (Poleshko et al., 2013). Taken together, these experiments indicate that membrane association of PRR14 is unlikely, and establish that positioning of PRR14 at the periphery is through Lamins A and/or C. Based on PRR14 heterochromatin localization in anaphase, we also proposed that PRR14 may have a mitotic ‘bookmarking’ role, specifying heterochromatin for return to the nuclear lamina at the end of mitosis when tethering is re-established (Poleshko and Katz, 2014).

Recent studies identified the *Caenorhabditis elegans* CEC-4 protein as a membrane-associated heterochromatin tether. CEC-4 encodes an HP1-like CD that interacts directly with methylated H3K9, therefore obviating the need for an HP1 adapter (Gonzalez-Sandoval et al., 2015). The identification of CEC-4 indicates that tethering using methylated H3K9 as anchoring points is conserved through evolution (Gonzalez-Sandoval et al., 2015; Harr et al., 2016; Kind et al., 2013; Towbin et al., 2013; van Steensel and Belmont, 2017). Thus far, only these three H3K9me-based tethers, LBR, PRR14 and CEC-4 have been identified. Being a non-membrane, nuclear lamina-associated protein, PRR14 is unique. However, a specific PRR14 domain that is responsible for PRR14 localization at the nuclear lamina has not been not identified.

Here, we have mapped a PRR14 nuclear lamina binding domain (LBD) (residues 231–351) that is both necessary and sufficient for nuclear lamina association, and also identified functional LBD core residues that are conserved beyond mammals. The discovery of a modular PRR14 LBD, in addition to the modular N-terminal HP1/heterochromatin binding domain, is consistent with the tethering function of PRR14. We also provide evidence that cycles of phosphorylation and dephosphorylation within the LBD contribute to PRR14 dynamics at the nuclear lamina. Furthermore, we identified a functional protein phosphatase 2A (PP2A) recognition motif (Hertz et al., 2016; Wang et al., 2016) as a core sequence within the highly conserved C-terminal Tantalus domain of PRR14 (residues 459–516). The overall findings provide key insights into the mechanism and evolutionary conservation of the PRR14 tether.

## RESULTS

### Identification of a minimal PRR14 domain that is sufficient for nuclear lamina association

**Fig. 1.**
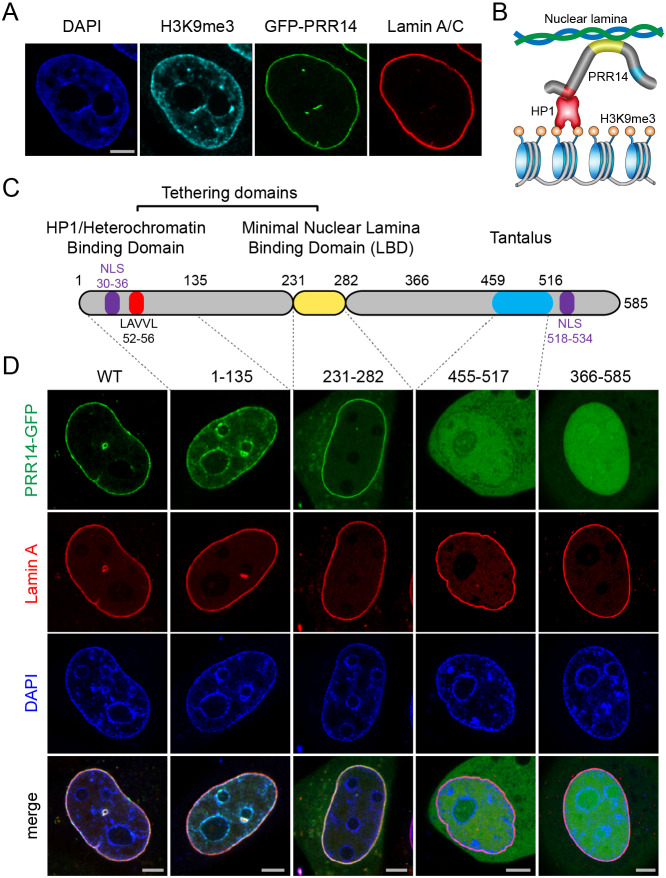
**Identification of a modular PRR14 nuclear lamina binding domain.** (A) Representative confocal imaging of HeLa cells showing localization of H3K9me3-marked heterochromatin (cyan), GFP-tagged full-length PRR14 (green), and Lamin A/C (red). (B) A schematic model of H3K9me3/HP1 heterochromatin tethering to the nuclear lamina via PRR14. See color scheme in panel C. (C) A schematic representation of the proposed PRR14 modular domain organization. Domains, LAVVL motif and nuclear localization sequences (NLS) are indicated. (D) Representative confocal images of live HeLa cells expressing mCherry–Lamin A transfected with GFP-tagged PRR14 fragments, as indicated (wild type, WT). The N-terminal fragment (1–135) shows localization to heterochromatin, the centrally located (231–282) fragment includes a surrogate SV40 NLS and is identified as a minimal nuclear lamina binding domain (LBD), the Tantalus domain (455–517) fragment localizes throughout the nucleoplasm and cytoplasm and the C-terminal fragment (366–585) localizes in the nucleoplasm. Scale bars: 5 µm. Images in A and D are representative of three experiments and >60 cells.

We showed previously that the N-terminal PRR14 1–135 region is necessary and sufficient for heterochromatin binding through a PRR14 LAVVL HP1/heterochromatin binding motif at positions 52–56 (Fig. 1) (Poleshko et al., 2013). Targeting of the PRR14 protein to the nucleus can occur via LAVVL-dependent HP1–heterochromatin binding during mitosis, and through nuclear localization signal (NLS) sequences at the N- and C-termini (Poleshko et al., 2013). Previously, we also found that the C-terminal portion of PRR14 (residues 366–585) was sufficient for localization to the nucleus via the C-terminal NLS, but this fragment did not localize to the nuclear lamina (Fig. 1C,D). When independently expressed, the highly conserved Tantalus protein family (Pfam; PF15386) domain (residues 459–516) shows no specific localization and is distributed throughout the whole cell (Fig. 1C,D). To determine which region(s) of PRR14 are required, or sufficient, for nuclear lamina association, a series of C-terminal truncations of the N-terminal GFP-tagged PRR14 protein were constructed (Fig. S1). Nuclear lamina localization was found to be retained for N-terminal fragments that included the first 272 residues, while nuclear lamina localization was lost with shorter truncations (Fig. S1A). With loss of nuclear lamina association, the residue 1–257, 1–241, 1–225 and 1–212 fragments appeared to localize to heterochromatin both in perinucleolar regions and at the nuclear periphery, similar to the localization of the 1–135 fragment (Fig. 1; Fig. S1). To validate this interpretation, a V54E and V55E double mutation was introduced in the LAVVL HP1/heterochromatin binding motif (residues 52–56) of the 1–324 and 1–288 constructs (which had apparent nuclear lamina localization), and the 1–212 construct (which had apparent heterochromatin localization) (Fig. S1B). Mutations in the HP1-binding motif had no effect on the localization of the 1–324 and 1–288 fragments to the periphery, whereas the 1–212 fragment became nucleoplasmic as expected due to the absence of both nuclear lamina and heterochromatin binding (Fig. S1B). The localization to the nuclear lamina of the 1–324 and 1–288 proteins with HP1-binding site mutations reinforces our previous interpretation that HP1/heterochromatin binding is not required for positioning of full-length PRR14 at the nuclear lamina.

Based on the retention of nuclear lamina association with the residue 1–272 fragment and loss of association with the residue 1–212 fragment, a 231–282 module was tested as a candidate LBD. This fragment lacked a nuclear targeting mechanism, resulting in cytoplasmic retention (data not shown). Addition of an N-terminal SV40 NLS improved nuclear localization of the 231–282 fragment. Strikingly, this 231–282 fragment localized at the nuclear periphery, associating with the nuclear lamina similarly to full-length PRR14 (Fig. 1D). Localization of the 231–282 fragment to the nuclear interior was confirmed using the differential permeability method (Fig. S2). These results identified the 231–282 fragment as a provisional, minimal lamina binding domain (LBD) (Fig. 1C).

### The PRR14 231–282 minimal LBD is evolutionarily conserved beyond mammals

**Fig. 2.**
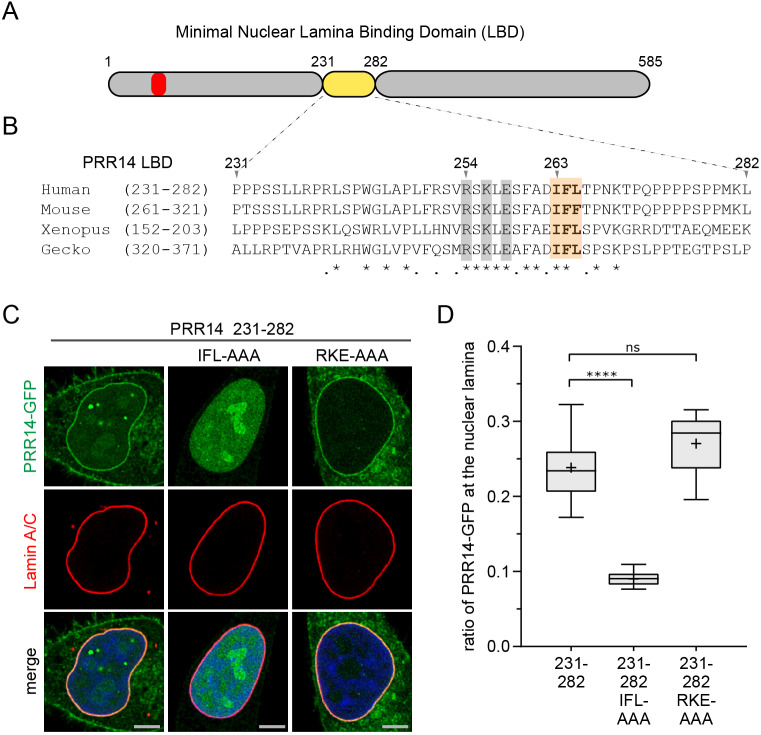
**Identification of functional, evolutionarily conserved residues in the 231–282 LBD.** (A) Diagram of PRR14 depicting the minimal 231–282 LBD. The LAVVL HP1/heterochromatin-binding motif (52–56) is indicated in red. (B) Protein sequence alignment of the human 231–282 PRR14 LBD with mouse, *Xenopus* and gecko orthologs identified conserved residues: charged (gray) and hydrophobic (orange). Amino acid sequence identity (*) and similarity (.) are indicated. (C) Representative confocal images of HeLa cells transfected with GFP-tagged PRR14 231–282 LBD containing the indicated amino acid substitutions. (D) Box plot demonstrating the proportion of the indicated PRR14-GFP proteins at the nuclear lamina compared to the total nuclear signal, calculated using Lamin A/C signal as a mask. Boxes indicate the interquartile range with the median represented by a horizontal bar. Whiskers are drawn using the Tukey method and + indicates the mean value. *n*=20 cells per condition. ****P<0.0001; ns, not significant (one-way ANOVA with Dunn's multiple comparison test). Scale bars: 5 µm.

Initial BLASTp analyses predicted that the PRR14 tether was unique to mammals (Poleshko et al., 2013). One difficulty in assessing the extent of evolutionary conservation of the PRR14 protein is the existence of the paralogous PRR14L protein (Table S1). The basis for assigning PRR14L as a human PRR14 paralog, despite their different sizes (2151 amino acids for PRR14L versus 585 amino acids for PRR14), is the highly conserved domain at the C-terminus of both proteins (positions 459–516 in PRR14 and positions 2026–2083 in PRR14L) which shows 74% identity and 79% similarity in this region. As mentioned (Fig. 1), this region has now been identified as a protein family domain (El-Gebali et al., 2019), Tantalus, which was defined by homology with the *Drosophila* Tantalus protein (Fig. 1C) (Aravind and Iyer, 2012; Dietrich et al., 2001; El-Gebali et al., 2019). Beyond this domain, the dissimilarity between mammalian PRR14, PRR14L and *Drosophila* Tantalus suggested that they have distinct functions. When we limited the BLASTp homology query to human PRR14 residues that target to the nuclear lamina, the LBD at positions 231–282, we found potential PRR14 orthologs in reptiles and amphibians (Fig. 2, Table S1). The mouse PRR14 LBD shares 94% sequence identity with the human PRR14 LBD (Fig. 2B), and as expected, the full-length mouse PRR14 protein localized to the nuclear lamina (Fig. S3A). The gecko lizard and *Xenopus* frog PRR14 proteins displayed 48% and 44% sequence identity and 59% and 55% percent sequence similarity, respectively, with the human PRR14 231–282 LBD. The full-length gecko and *Xenopus* candidate PRR14 orthologs are larger than the 585-amino-acid human PRR14 (727 and 699 amino acids, respectively), and show very limited homology overall with the human PRR14, outside of the Tantalus domain (82% and 70% identity to human Tantalus, respectively) (Table S1). As further evidence of the functional conservation of the minimal LBD, the full-length human PRR14 was found to localize to the nuclear lamina in *Xenopus laevis* cells (Fig. S3A). To confirm functionalities of the LBD fragments, the minimal human and *Xenopus* LBDs were introduced into *Xenopus* cells, and were found to localize to the nuclear lamina (Fig. S3B). Similarly, the LBD from the putative *Xenopus* PRR14 protein localized to the nuclear lamina in human cells (Fig. S3B). As in Fig. S2, we carried out differential antibody accessibility analysis to confirm that the *Xenopus* GFP-tagged LBD localized inside the nucleus of HeLa cells (data not shown).

Having shown common functionality of non-mammalian LBDs, the alignment of the four LBD sequences was next used to predict key functional residues (Fig. 2A,B; Fig. S3C). A provisional core homology region in the minimal human PRR14 LBD was identified at positions 254–265 (RSKLESFADIFL) (Fig. 2B; Fig. S3C, Table S1), and independent sets of alanine substitutions were introduced for the charged residues R, K and E, and for the hydrophobic residues I, F and L. A triple alanine substitution, RKE–AAA, had no effect on localization of the minimal human PRR14 231–282 LBD, while triple alanine substitution of the IFL residues with AAA resulted in dramatic loss of nuclear lamina localization of the LBD (Fig. 2C,D). The effects of these substitutions were measured as a ratio of the GFP signal at the nuclear periphery to total signal in the nucleus (see Materials and Methods section for details). These results were consistent with the loss of nuclear lamina localization observed for the truncated PRR14 1–257 residue fragment, which retains the RKE residues but was missing the IFL motif (Figs S1,S3C).

### The minimal PRR14 231­–282 LBD and conserved residues therein are required for efficient nuclear lamina localization of full-length PRR14

**Fig. 3.**
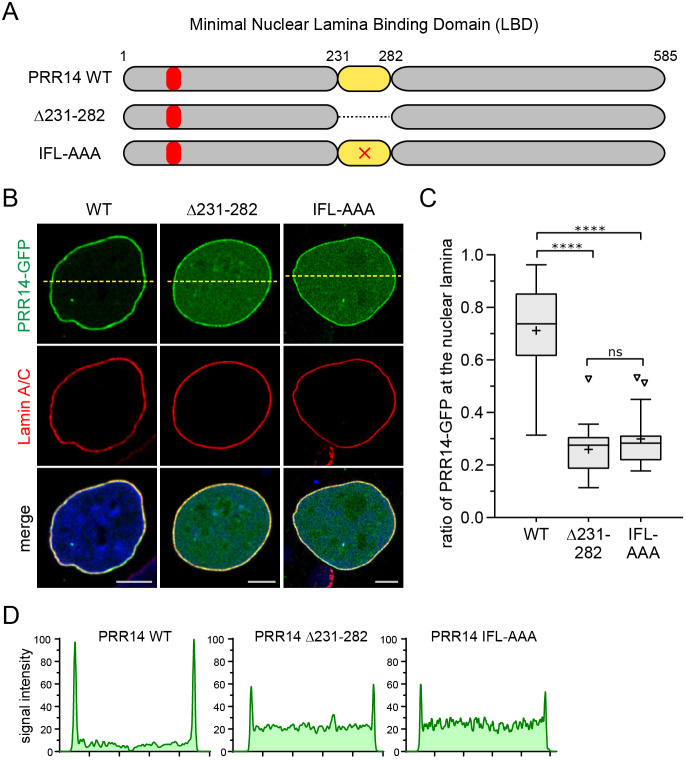
**The 231–282 LBD is required for efficient localization of PRR14 to the nuclear lamina.** (A) Diagram of human wild-type (WT) and mutated PRR14 depicting the 231–282 LBD, Δ231–282, and IFL to AAA substitution (×) in the conserved core sequence. The LAVVL HP1/heterochromatin-binding motif (52–56) is indicated in red. (B) Representative confocal images of HeLa cells transfected with the GFP-tagged PRR14 constructs (green) depicted in A, with anti-Lamin A/C staining (red) and DAPI counterstaining. Dashed lines indicate line sections for signal intensity profiles shown in D. (C) Box plot demonstrating the proportion of indicated PRR14–GFP protein at the nuclear lamina compared to the total nuclear signal, calculated using Lamin A/C signal as a mask. Boxes indicate the interquartile range with the median represented by a horizontal bar. Whiskers are drawn using the Tukey method and + indicates the mean value. *n*=20 cells per condition. (D) Line signal intensity profiles of sections indicated by dashed lines in B. ****P<0.0001; ns, not significant (one-way ANOVA with Dunn's multiple comparison test). Scale bars: 5 µm.

As a rigorous test of whether the minimal PRR14 231–282 LBD was solely responsible for targeting of PRR14 to the nuclear lamina, we constructed a PRR14 deletion mutant (Δ231–282). As shown in Fig. 3, deletion of the 231–282 region resulted in significant, but incomplete loss of nuclear lamina localization. Moreover, the IFL to AAA substitution was sufficient to produce a similar loss in nuclear lamina localization (Fig. 3B–D). To assess whether the residual localization at the nuclear periphery was due to binding to peripheral heterochromatin, V54E and V55E mutations were introduced in the LAVVL HP1/heterochromatin binding motif (positions 52–56) in the context of the Δ231–282 and IFL to AAA mutants. Heterochromatin binding was assessed in mouse cells that feature H3K9me3/HP1-rich chromocenters that decorate the nucleoli and their periphery, as well as a peripheral layer of heterochromatin (Eberhart et al., 2013; Politz et al., 2013). As shown in Fig. S4, the composite mutants lost heterochromatin localization as expected, but retained residual peripheral localization. These results suggested that the minimal 231–282 LBD is required for efficient nuclear lamina localization, but also indicated that additional sequences were contributing to nuclear lamina localization. However, extending the deletion on the N-terminal side (Δ135–282) did not impact the residual nuclear lamina binding (data not shown).

### Identification of an optimal, modular nuclear lamina binding domain

**Fig. 4.**
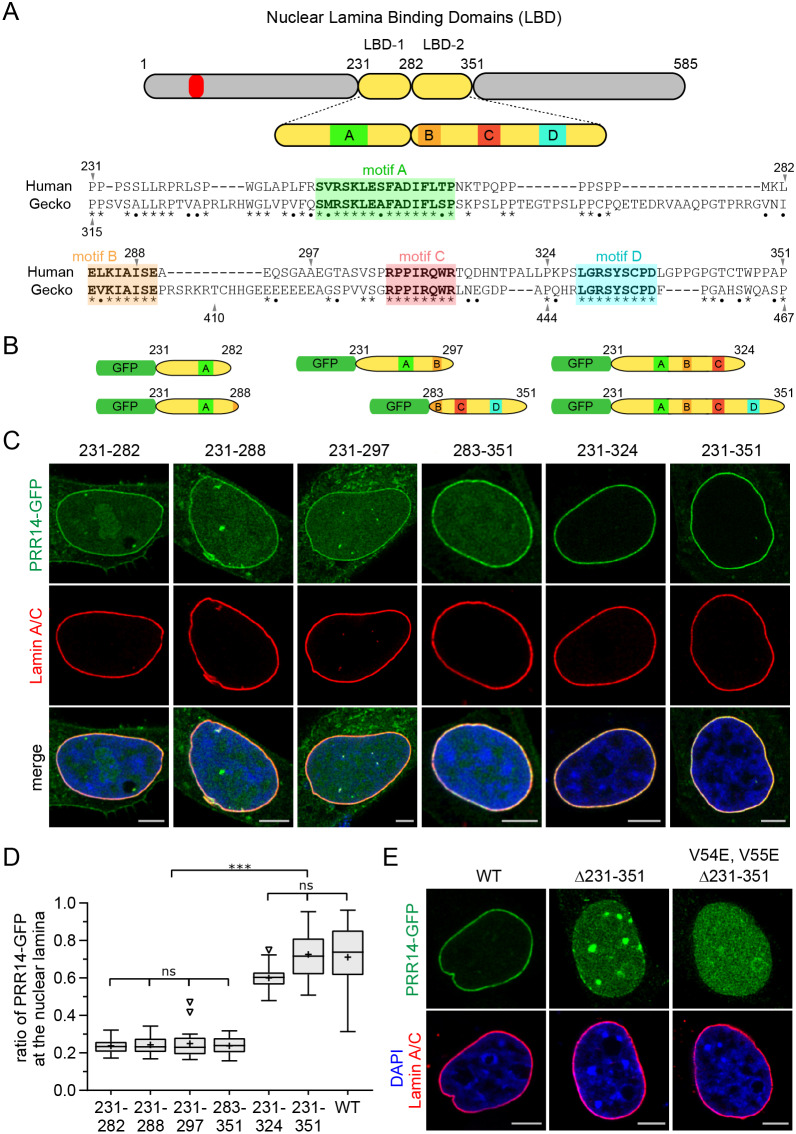
**Identification of an optimal PRR14 LBD module.** (A) Diagram and sequence conservation of the region downstream of the 231–282 LBD hypothesized to contribute to nuclear lamina binding. The LAVVL HP1/heterochromatin-binding motif (52–56) is indicated in red. Sequence alignment of human and gecko PRR14 identified candidate functional motifs. Amino acid sequence identity (*) and similarity (•) are indicated. Conserved motifs are designated as A through D. (B) Schematic representation of PRR14 LBD constructs with conserved motifs indicated as in A. All constructs included an SV40 NLS. (C) Representative confocal images of HeLa cells transfected with the indicated N-GFP-tagged PRR14 fragments. Independent of the 231–282 LBD, the 283–351 fragment was found to localize to the nuclear lamina as a second independent modular LBD domain (LBD-2). (D) Box plot demonstrating the proportion of the indicated PRR14–GFP proteins, as described in panel B, at the nuclear lamina compared to the total nuclear signal, calculated using Lamin A/C signal as a mask (wild type, WT). Boxes indicate the interquartile range with the median represented by a horizontal bar. Whiskers are drawn using the Tukey method and + indicates the mean value. *n*=20 cells per condition. The 231–324 fragment was found to have optimal nuclear lamina binding. (E) Representative confocal images of HeLa cells transfected with the indicated N-GFP-tagged PRR14 constructs: wild type (WT), 231–351 deletion and a composite mutant with the 231–351 deletion and substitutions in the LAVVL sequence (V54E, V55E) required for heterochromatin binding. ***P<0.001; ns, not significant (one-way ANOVA with Dunn's multiple comparison test). Scale bars: 5 µm. Images in E are representative of three experiments and >60 cells.

The residual nuclear lamina binding of the Δ231–282 mutant (Fig. 3) forced us to reassess whether the 231–282 region solely accounted for nuclear lamina targeting. We considered that the lamina binding domain might extend further to the C-terminal side of the LBD. Alignment of human and gecko PRR14 sequences revealed several conserved downstream motifs (denoted B, C, D) that were candidates for contributing to nuclear lamina binding, along with motif A within the minimal 213–282 LBD (Fig. 4A). A human PRR14 231–351 fragment harboring all downstream conserved motifs showed nuclear lamina association that was quantitatively indistinguishable from full-length wild-type PRR14 (Fig. 4B–D). To determine the role of motifs B, C and D, fragments encompassing positions 231–288, 231–297, 231–324, and 283–351 were designed (Fig. 4B). The 231–324 fragment again showed nuclear lamina association that was not significantly different from the full-length protein, indicating that motifs B and/or C were important for nuclear lamina localization (Fig. 4B–D). The 231–288 and 231–297 fragments that contain motifs A and B showed weaker association compared to the 231–324 fragment, similar to the minimal 231–282 LBD. Surprisingly, the PRR14 283–351 fragment containing only motifs B and C independently localized to the nuclear lamina and was therefore denoted LBD-2 (Fig. 4A–D). The 231–282 fragment was re-designated LBD-1. To investigate the biological relevance of these motifs, three equivalent fragments from the putative gecko PRR14 protein were designed encompassing motifs A alone, A and B, or A, B, and C (residues 320–371, 320–410 and 320–444 for A, B and C, respectively). Remarkably, all three gecko PRR14 fragments localized to the nuclear lamina in HeLa cells, with the 320–444 fragment (equivalent of the human 231–324 fragment) being most efficient (Fig. S5). These results indicate that the mechanism of PRR14 nuclear lamina binding is conserved in vertebrate evolution, and that motifs A and C are required for optimal binding.

Having defined an extended modular LBD comprised of LBD-1 and LBD-2, we constructed a larger deletion in human PRR14 (Δ231–351), which was found to completely disable nuclear lamina binding (Fig. 4E) compared to Δ231–282 (Fig. 3B,C; Fig. S4). Lacking nuclear lamina binding, the Δ231–351 protein appeared to relocate to heterochromatic foci (Fig. 4E). A composite HP1-binding mutant, with the V54E and V55E mutations introduced into Δ231–351, was found throughout the nucleoplasm, indicating lack of both heterochromatin and nuclear lamina binding (Fig. 4E). In addition, we confirmed that the 231–351 LBD fragment did not associate with heterochromatin, thus eliminating such a mechanism in peripheral localization for LBD-1 or LBD-2 (Fig. S6). These results are consistent with previous observations that the PRR14 V54E, V55E mutant lacks heterochromatin binding (Poleshko et al., 2013). Taken together, our data indicate that the 231–324 PRR14 fragment is sufficient and optimal for nuclear lamina binding. These findings support the known tethering function of PRR14 by defining a modular lamina binding domain (LBD) capable of mediating bridging between the nuclear lamina and heterochromatin.

### Evidence for regulation of PRR14­–nuclear lamina association through LBD phosphorylation

Mitotic entry is accompanied by numerous phosphorylation events that trigger nuclear envelope and nuclear lamina disassembly (Dephoure et al., 2008). Such phosphorylation is catalyzed by a mitotic serine-threonine cyclin-dependent kinase (CDK), and the majority of phosphorylation sites conform to the consensus sequences serine-proline (SP) and threonine-proline (TP). We found previously that PRR14 is disassembled from the intact nuclear lamina during early mitosis (Poleshko et al., 2013). We therefore searched the PRR14 protein sequence for SP and TP sites, and also examined PhosphositePlus (Hornbeck et al., 2015) and ProteomicsDB (Schmidt et al., 2017) databases for evidence of *in vivo* SP and TP phosphorylation sites within the LBD that could regulate nuclear lamina association. In addition, we repurposed our data from a BioID-based (Roux et al., 2012) search for PRR14 partners, where we could examine phosphorylation of the PRR14 bait protein (data not shown). As shown in Table S2, we identified four *in vivo* phosphorylation sites in an [S/T]P sequence context within the 231–282 LBD-1 at positions S242, T266, T270 and S277. All four [S/T]P sites were mutated in a variety of cancers (Table S2). Of note, no additional phosphorylation sites were detected in the LBD-2 fragment (residues 283–351).

**Fig. 5.**
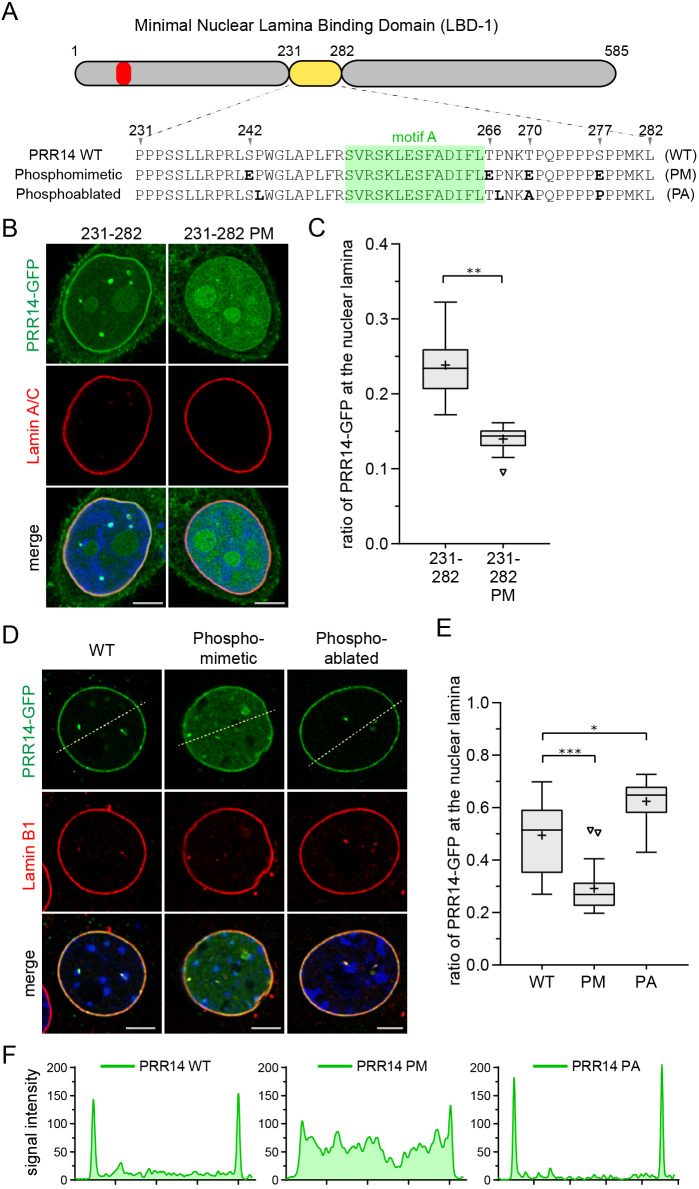
**LBD phosphomimetic and phosphoablation substitutions affect PRR14–nuclear lamina association.** (A) Diagram and sequence of the human PRR14 231–282 LBD highlighting serine/threonine [S/T]P phosphorylation sites at positions 242, 266, 270 and 277 in wild type (WT). Phosphomimetic (PM) and phosphoablation (PA) substitutions are shown. The LAVVL HP1/heterochromatin-binding motif (52–56) is shown in red. (B) Representative confocal images of HeLa cells expressing the GFP-tagged PRR14 LBD (231–282) with phosphomimetic (PM) glutamic acid substitutions, showing loss of nuclear lamina localization. (C) Box plot demonstrating the proportion of indicated PRR14-GFP proteins from panel B at the nuclear lamina compared to the total nuclear signal, calculated using Lamin A/C signal as a mask. Boxes indicate the interquartile range with the median represented by a horizontal bar. Whiskers are drawn using the Tukey method and + indicates the mean value. *n*=20 cells per condition. (D) Representative confocal images of C2C12 cells expressing phosphomimetic (PM) and phosphoablation (PA) mutants of GFP-tagged full-length PRR14. Dashed lines indicate line sections quantified in F. (E) Box plot demonstrating the proportion of the indicated PRR14–GFP proteins from panel D at the nuclear lamina compared to the total nuclear signal, calculated using the Lamin B1 signal as a mask. Boxes indicate the interquartile range with the median represented by a horizontal bar. Whiskers are drawn using the Tukey method and + indicates the mean value. *n*=20 cells per condition. (F) Line signal intensity profiles corresponding to dashed lines in panel D. ***P<0.001; **P<0.01; *P<0.05 (one-way ANOVA with Dunn's multiple comparison test). Scale bars: 5 µm.

We substituted each of the four S/T residues with a phosphomimetic residue, glutamic acid, in the context of the GFP-tagged 231–282 LBD fragment (Fig. 5A). This resulted in a dramatic loss of nuclear lamina localization, with accumulation in the nucleoplasm (Fig. 5B,C). Introduction of the same four phosphomimetic substitutions into the full-length human PRR14 protein produced a similar loss of nuclear lamina association (Fig. 5D–F). Phosphomimetic substitutions are not always fully effective at reproducing the phosphorylated state (Dephoure et al., 2013), and therefore it is difficult to assess whether additional mechanisms account for the observed residual nuclear lamina retention. Next, we created a quadruple PRR14 mutant bearing four cancer-associated missense mutations that ablate the [S/T]P consensus sites (P243L, T267L, T270A and S277P). This quadruple PRR14 mutant, designed to block phosphorylation, showed an unexpected and significant phenotype: higher accumulation at the nuclear lamina during interphase than wild-type PRR14 protein (Fig. 5D–F).

### PRR14 dynamically associates with the nuclear lamina during interphase

**Fig. 6.**
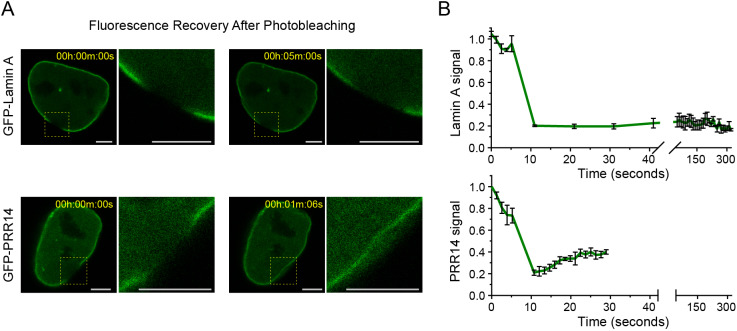
**FRAP analysis showing that PRR14 is mobile at the nuclear lamina.** HeLa cells were transfected with GFP-tagged Lamin A or GFP-tagged PRR14. A region of interest at the nuclear lamina was photobleached and fluorescence recovery was monitored. (A) Representative confocal images of GFP–PRR14 or GFP–Lamin A after photobleaching and recovery. Scale bars: 6 µm. (B) Graphs show GFP signal intensity monitored over a 5 min period. Data analyzed using the EasyFrap software indicated a PRR14 recovery half time of 6.4 s with an R-squared value of 0.95. Data are mean±s.e.m. of three experiments.

The finding that ablation of the PRR14 LBD-1 phosphorylation sites resulted in higher affinity for the nuclear lamina suggested that phosphorylation might regulate dynamic association of PRR14 with the nuclear lamina during interphase. To measure the mobility of PRR14 at the nuclear lamina, we used a conventional fluorescence recovery after photobleaching (FRAP) approach (Goldman et al., 2002). Cells were transfected with either GFP–PRR14 or GFP–Lamin A. Peripheral regions-of-interest were laser bleached and the recovery time was monitored. As a component of the nuclear lamina framework, Lamin A was quite stable over the measured 5 min period (Fig. 6; Movie 1), as observed previously (Goldman et al., 2002). In contrast, PRR14 was found to exchange rapidly at the nuclear lamina, with a recovery half time of 6.4 s (Fig. 6; Movie 2). Taken together with the quadruple phosphoablation and phosphomimetic results, one interpretation of these findings is that the rapid exchange of PRR14 at the nuclear lamina is phosphoregulated, with phosphorylation of the LBD triggering release.

### PP2A phosphatase binds PRR14 via a motif in the conserved Tantalus domain and may regulate PRR14 association with the nuclear lamina

**Fig. 7.**
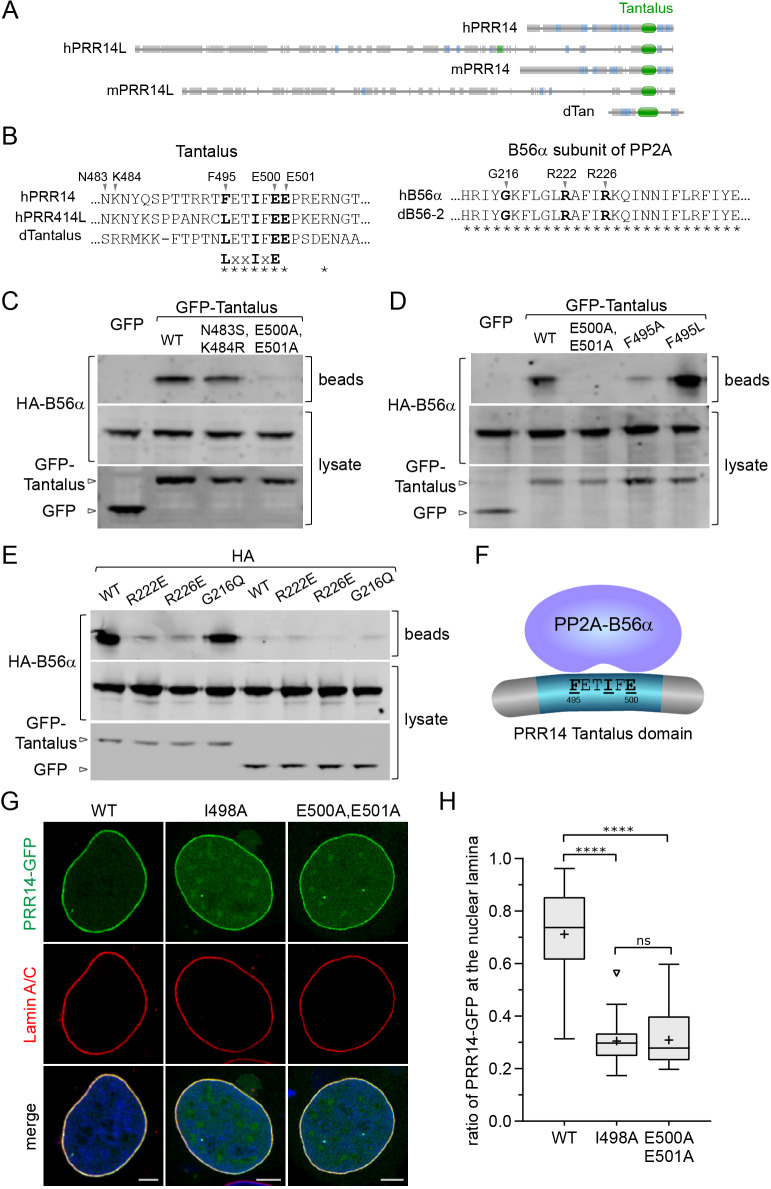
**PP2A binds PRR14 and regulates PRR14–nuclear lamina association.** (A) Diagrams of indicated proteins showing Tantalus domain location. (B) Summary of key residues in the PP2A B56α subunit and PRR14 Tantalus domain that were predicted to mediate B56α binding to the corresponding PRR14 [L/F/M]xxIxE motif. Amino acid sequence identity (*) is indicated. (C) Anti-GFP beads were added to lysates of HeLa cells that had been co-transfected with HA-tagged B56α, and free GFP or GFP fused to the PRR14 Tantalus domain. The indicated Tantalus substitutions were tested for effects on B56α interactions, alongside the wild type (WT). Bead-bound proteins were analyzed by western blotting using anti-HA antibodies to detect B56α–PRR14 Tantalus interactions. Anti-GFP was used to monitor GFP/GFP-Tantalus in the lysates. The E500A, E501A substitution inhibited the interaction as predicted. (D) Using an experimental design as described in C, PRR14 Tantalus domain substitutions E500A, E501A and F495A were found to inhibit binding, whereas F495L increased binding. (E) Using an experimental design as described in C, HA-tagged B56α subunits harboring the indicated amino acid substitutions were assayed for binding to the GFP–Tantalus domain. (F) A schematic diagram showing PP2A interaction with the PRR14 Tantalus domain. (G) Representative confocal images of HeLa cells transfected with the indicated Tantalus domain amino acid substitutions introduced into full-length GFP-tagged human PRR14 (see panel B). The indicated substitutions resulted in reduced nuclear lamina association. (H) Box plot demonstrating the proportion of the indicated PRR14–GFP proteins, as depicted in G, at the nuclear lamina compared to the total nuclear signal, calculated using Lamin A/C signal as a mask. Boxes indicate the interquartile range with the median represented by a horizontal bar. Whiskers are drawn using the Tukey method and + indicates the mean value. *n*=20 cells per condition. ****P<0.0001; ns, not significant (one-way ANOVA with Dunn's multiple comparison test). Scale bars: 5 µm. Data shown in C–E are representative of more than one experiment.

Protein-partner screens had detected PRR14, using either HP1 (Nozawa et al., 2010; Rual et al., 2005) or phosphatase PP2A (Herzog et al., 2012) as bait. The latter finding implicated PRR14 as a substrate or partner (or both) for PP2A. PP2A is a complex trimeric serine/threonine phosphatase, composed of three subunit types: catalytic, structural and regulatory (Shi, 2009; Virshup and Shenolikar, 2009). Human PRR14, PRR14L and *Drosophila* Tantalus protein share the Tantalus domain and bind PP2A complexes (Glatter et al., 2009; Guruharsha et al., 2011; Herzog et al., 2012) (Fig. S7). Recently, a common motif was identified among PP2A substrates that is recognized by the PP2A-B56α regulatory subunit (also known as PPP2R5A in humans) (Hertz et al., 2016; Wang et al., 2016). We found that this short linear motif (SLiM), [L/F/M]xxIxE (Hertz et al., 2016), corresponds to the most conserved portion of the Tantalus domain – [L/F]ETIFE (Fig. 7; Fig. S7, Table S1). To test whether the PRR14 Tantalus domain could bind the PP2A-B56α subunit, we used co-transfection pulldown and mutagenesis experiments with a human PRR14 Tantalus domain construct (455–517) and the human B56α subunit (Fig. 7B–E). The GFP-tagged Tantalus domain was able to pull down B56α, with non-fused GFP serving as a negative control (Fig. 7C). Next, amino acid substitutions were introduced into the PRR14 FETIFE motif. The [L/F/M]xxIxE consensus motif frequently features additional acidic residues on the C-terminal side, as in the case of the PRR14 motif FETIFE(E) (Fig. 7B). Substitution with alanine of the predicted key EE residues (Hertz et al., 2016) of the PRR14 Tantalus motif resulted in a loss of binding to B56α (Fig. 7B–D). Substitution of two non-conserved residues (N483S, K484R) served as a negative control and had no effect (Fig. 7C). An F to A mutation in the first position of the PRR14 Tantalus FETIFE largely disabled binding, while an F to L substitution resulted in tighter binding to B56α, as predicted (Fig. 7D) (Hertz et al., 2016). Disruptive mutations in the B56α HEAT repeat binding pocket that engages the [L/F/M]xxIxE motif (R222E, R226E) resulted in loss of Tantalus binding (Fig. 7E), whereas a negative control G216Q substitution had no effect (Hertz et al., 2016). As noted above, the *Drosophila* Tantalus protein (dTantalus) was found to interact with the *Drosophila* B56α PP2A subunit (dB56-2, also known as Wdb) (Guruharsha et al., 2011). It is predicted that this interaction is mediated by the dTantalus LETIFE motif and the highly conserved binding pocket region of dB56-2 (Fig. 7B). There is no evidence that the small dTantalus protein functions as a tether, but rather may simply share with PRR14 the Tantalus domain and PP2A-binding motif.

We hypothesized that PP2A regulates PRR14 localization at the nuclear lamina by dephosphorylating S/T residues in the LBD. It was therefore predicted that disabling the PRR14 FETIFE motif would result in loss of PP2A binding to PRR14 and increased phosphorylation of the PRR14 LBD, which would lead to reduction of PRR14–nuclear lamina association. As shown in Fig. 7G,H, this was indeed the case, as tested with two independent mutations (I498A and E500A, E501A). Kinase and PP2A phosphatase activities are thereby implicated in mediating dynamic association of PRR14 with the nuclear lamina.

## DISCUSSION

### PRR14 tethers H3K9me3-marked heterochromatin to the nuclear periphery via targeting to the nuclear lamina

There is considerable interest in understanding how the heterochromatin compartment is organized at the inner nuclear periphery, and how lineage-inappropriate genes are silenced through such positioning (Buchwalter et al., 2019; Shevelyov and Ulianov, 2019; van Steensel and Belmont, 2017; Wong and Reddy, 2015). Emerging findings have pointed to an evolutionarily conserved mechanism whereby a class of ‘tethering proteins’ function to organize H3K9me-modified heterochromatin at the nuclear envelope (Gonzalez-Sandoval et al., 2015; Harr et al., 2016; Poleshko et al., 2013, 2019; Towbin et al., 2013; van Steensel and Belmont, 2017). A common element of this class of tethers is that H3K9me heterochromatin modifications serve as anchoring points for attachment of heterochromatin to the nuclear envelope. Two members of this class, LBR (Olins et al., 2010) and CEC-4 (Gonzalez-Sandoval et al., 2015) anchor heterochromatin to nuclear membranes, and their roles in heterochromatin organization, as well as cell differentiation have been described (Gonzalez-Sandoval et al., 2015; Solovei et al., 2013).

**Fig. 8.**
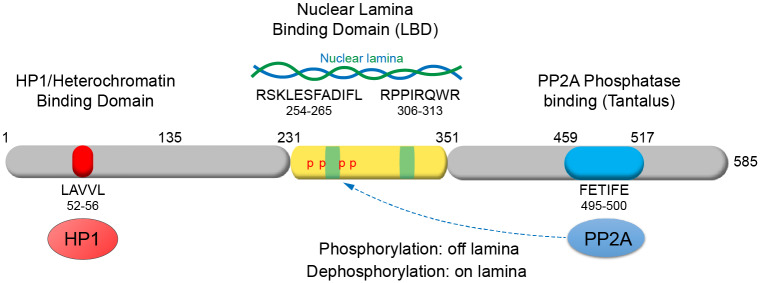
**A schematic model of the modular organization of the PRR14 functional domains.** PRR14 is found to be a highly modular protein. Several short evolutionarily conserved motifs play key roles in bivalent tethering between heterochromatin and the nuclear lamina, and are involved in regulation of association with the nuclear lamina.

We previously identified the human PRR14 protein as an epigenetic repressor and determined that it functions as a heterochromatin tether through HP1 and H3K9me3 (Poleshko et al., 2013). However, unlike the membrane-associated LBR and CEC-4 proteins, PRR14 is a non-membrane protein and associates with the nuclear lamina through Lamin A/C (Poleshko et al., 2013). PRR14 is thereby expected to be more mobile than the membrane-bound LBR (Giannios et al., 2017) and CEC-4 proteins. Here we demonstrate that PRR14 contains a modular nuclear lamina binding domain (LBD). Combined with existence of the PRR14 heterochromatin binding domain (amino acids 1–135), these findings represent the first example of a modular mechanism through which HP1 and H3K9me3-marked heterochromatin can be tethered to the nuclear lamina, rather than the inner nuclear membrane (Fig. 8).

As noted above, we previously demonstrated that Lamin A/C knockdown is sufficient to release PRR14 from the nuclear periphery (Poleshko et al., 2013). Furthermore, PRR14 does not localize with membranes during mitosis, whereas LBR does (Poleshko et al., 2013). Although PRR14 requires Lamin A/C for peripheral localization, we have not yet determined whether PRR14 is among the proteins that interact directly with Lamin A or Lamin C (Simon and Wilson, 2013). However, PRR14 was recently identified as a candidate nuclear lamina binding protein in live cells using BioID (Cutler et al., 2019 preprint), and our own BioID analysis also detected Lamin A/C as a candidate partner of PRR14 (data not shown).

Regarding how the unusually high proline content of PRR14 might contribute to tethering function, we previously presented structure-prediction analysis suggesting that the PRR14 protein contains disordered regions (Poleshko et al., 2013). Our recent experimental results suggest that PRR14 might adopt an extended rod-like structure in live cells (data not shown). It is possible that the high proline content may promote this extended structure, and thereby contribute to the function of PRR14 as a tether. Furthermore, SLiMs that mediate protein-protein interactions, such as the PP2A recognition motif (Hertz et al., 2016), are generally found in disordered regions. It is possible that PRR14 functions as a disordered scaffold, linking heterochromatin to the nuclear lamina through conserved peptides, thus fulfilling a hallmark of intrinsically disordered proteins. The binding of PP2A to the PRR14 scaffold via the well characterized SLiM might signify recruitment of PP2A not only to the PRR14 substrate, but to other neighboring protein substrates as well. Interestingly, the predicted disorder feature of PRR14 might be shared with the nuclear lamina-binding, nuclear membrane protein emerin (Berk et al., 2014; Samson et al., 2016). It is possible that this characteristic will emerge as being relevant to specific functions of proteins that localize to the nuclear lamina/nuclear membrane.

### Mapping of an optimal PRR14 LBD as guided by evolutionary conservation

We initially identified an autonomous nuclear lamina binding domain (LBD-1) of PRR14 that maps between positions 231–282 and is sufficient for localization to the nuclear lamina (Fig. 1; Fig. S1). This region includes a core motif (motif A, positions 254–265) that pointed to functional conservation beyond mammals (Fig. 2; Table S1). Deletion of the 231–282 fragment, or substitutions within the core motif, resulted in dramatic, but incomplete loss of nuclear lamina localization of the full-length PRR14 protein (Fig. 3; Fig. S4). Residual localization of the Δ231–282 PRR14 at the nuclear lamina suggested that additional PRR14 regions participate in nuclear lamina association. Analysis of the evolutionary conservation of PRR14, and additional experiments, revealed a second, contiguous, region of PRR14 (positions 283–351) that localizes autonomously to the nuclear lamina (denoted LBD-2) (Fig. 4). As expected, deletion of both autonomous LBDs (Δ231–351) resulted in complete loss of PRR14 nuclear lamina localization (Fig. 4E). At the same time, the 231–351 PRR14 fragment localized to the nuclear lamina with similar efficiency as full-length PRR14. Combined, these results demonstrate that the 231–351 fragment (denoted the PRR14 LBD) alone mediates association with the nuclear lamina (Fig. 8). Within the 231–351 PRR14 LBD domain we identified four evolutionarily conserved motifs, and demonstrated that inclusion of motif A (positions 254–265) and motif C (positions 306–313) is necessary for nuclear lamina targeting of the LBD (Figs 4,8). We also demonstrated that heterochromatin binding and nuclear lamina binding can be completely uncoupled (Fig. S6). The results of these rigorous experiments demonstrated the existence of a modular domain (positions 231–351) that solely accounts for nuclear lamina targeting of PRR14. The results from mapping of the reptilian PRR14 LBD (Fig. 4A; Fig. S5) further support this conclusion. The identification of this reptilian LBD, which localizes to the nuclear lamina in human cells, points to an evolutionarily conserved targeting mechanism. As nuclear lamins are highly conserved in evolution, the interspecies functionality may reflect a direct interaction between the reptilian LBD and the human nuclear lamins.

### Evidence that localization of PRR14 at the nuclear lamina is regulated by phosphorylation

A general paradigm is that phosphorylation promotes disassembly of nuclear envelope components during mitosis, and that dephosphorylation is required for nuclear reassembly (Dephoure et al., 2008). Consistent with this, we previously showed that PRR14 detaches from the nuclear lamina in prophase (Poleshko et al., 2013). However, it has been reported that nuclear lamins are rapidly phosphorylated and dephosphorylated during interphase, indicating that phosphoregulation of nuclear envelope components is not limited to mitosis (Kochin et al., 2014). We provide evidence that PRR14 association with the nuclear lamina is regulated through phosphorylation of [S/T]P sites within the 231–282 LBD-1 region (Fig. 5A–C). Specifically, we demonstrated that PRR14 LBD-1 phosphomimetic and phosphoablation mutants (Fig. 5D–F) have weaker and stronger association with the nuclear lamina, respectively. Independently, our FRAP analysis demonstrated rapid recovery of PRR14 at the nuclear lamina, suggesting that PRR14 dynamically associates with the nuclear lamina during interphase (Fig. 6). We hypothesize that such dynamics are regulated by phosphorylation. However, future studies will be required to obtain more direct evidence for phosphoregulation of dynamic PRR14–nuclear lamina interactions during interphase.

We also found that a highly conserved motif within the PRR14 Tantalus domain corresponds to a functional PP2A phosphatase recognition motif (Fig. 7). Mutation of this motif, expected to prevent PP2A binding, promoted release of PRR14 from the nuclear lamina during interphase. As the free Tantalus domain has no obvious direct role in targeting to the nuclear lamina (Fig. 1C,D), this finding is consistent with loss of PP2A binding resulting in an increase in LBD phosphorylation thereby leading to release from the nuclear lamina as predicted from phosphomimetic experiments (Fig. 5). Taken together, our results support roles for phosphorylation of the LBD, and dephosphorylation by PP2A, in regulating dynamic PRR14–nuclear lamina association during interphase. Further studies will be required to provide more direct evidence that PP2A acts on phosphorylation sites in the LBD.

The dynamic nature of PRR14 suggests that this tether may facilitate observed exchanges of heterochromatic loci between the peripheral and perinucleolar heterochromatin compartments (Kind et al., 2013; van Koningsbruggen et al., 2010; van Steensel and Belmont, 2017; Vertii et al., 2019). These peripheral–perinucleolar exchanges are presumed to take place during mitosis, but a dynamic tether could facilitate more rapid exchanges during interphase. Alternatively, PRR14 mobility may simply reflect its association with the HP1 protein, which has been reported to form phase-separated droplets (Strom et al., 2017; Tatarakis et al., 2017).

### Insights into the function of PRR14L

PRR14 and its paralog PRR14L primarily share the Tantalus domain, including the B56α binding motif (Fig. 7A; Fig. S7, Table S1). Recent studies identified PRR14L as a disease gene, driving age-related clonal hematopoiesis and contributing to myeloid neoplasia (Chase et al., 2019). These authors concluded, as we have, that the paralogous relationship between PRR14 and PRR14L is defined solely by the common C-terminal Tantalus domain. They also found that PRR14L localizes to the midbody, suggesting a role in cell division. We suggest further that the larger PRR14L protein has no function in heterochromatin organization or nuclear lamina binding, but rather may function as a scaffold for phosphatase PP2A at the midbody.

### The optimal PRR14 LBD can be used to target fusion proteins to the nuclear lamina

Our findings show that the 94-amino-acid 231–324 LBD fragment containing the evolutionarily conserved motifs A and C (Fig. 8) effectively promotes localization of GFP to the nuclear lamina. This optimal LBD fragment may therefore be used as a tag for targeted localization of proteins to the nuclear periphery. One obvious use would be to fuse enzymatically disabled Cas9 to the LBD to deliver genes to the nuclear periphery using locus-specific guide RNAs (Lyu and Corces, 2019; Wang et al., 2018). In contrast to current fusion protein strategies that target the inner nuclear membrane, a PRR14 LBD fusion protein will remain soluble and perhaps more effectively deliver genes to the repressive heterochromatin–nuclear lamina compartment. The optimal PRR14 LBD may therefore be useful as part of the recently described ‘toolbox’ of such targeting domains (Wang et al., 2018).

### Summary

PRR14 was found previously to function as a heterochromatin–nuclear lamina tether, and here we show that PRR14 encodes a modular, evolutionarily conserved nuclear lamina binding domain. These findings reinforce the tethering model by demonstrating a mechanism by which PRR14 localizes to the nuclear lamina. We also provide evidence that the PRR14 tether exchanges rapidly at the nuclear lamina through phosphoregulation of the LBD, and that phosphatase PP2A plays a role in this process. Further study of tethering proteins will likely contribute to an understanding of the mechanisms that underlie heterochromatin disorganization in cancer and aging.

## MATERIALS AND METHODS

### Cells

Murine C2C12 skeletal myoblast and HeLa cells were obtained from the American Type Culture Collection (ATCC, cat# CRL-1772 and cat# CCL-2) and tested negative for mycoplasma contamination. A second lot of HeLa cells were obtained from Tim Yen (Fox Chase Cancer Center) and also tested negative for mycoplasma. C2C12 and HeLa cells were maintained at 37°C in DMEM supplemented with 10% FetalPlex serum complex (Gemini, cat# 100-602), penicillin and streptomycin. Alternatively, HeLa cells were grown in DMEM containing 10% FBS, supplemented with penicillin-streptomycin (Corning, cat# 30-002-CI) and Fungizone (ThermoFisher, cat# 15290-018). *X. laevis* S3 cells were a gift from Mathew Good, University of Pennsylvania School of Medicine and were grown in L-15 medium (GIBCO) supplemented with 10% FBS, sodium pyruvate and penicillin-streptomycin at 27°C.

### Plasmids

A human PRR14 expression vector was obtained previously from OriGene Technologies, encoding a C-terminal fusion with TurboGFP (cat# RG208696). The human PRR14 ORF was transferred to N-terminal mGFP and mRFP vectors from OriGene (pCMV6-AN-mGFP, cat# PS100048; pCMV6-AN-mRFP, cat# PS100049) via the Origene PrecisionShuttle system using SgfI and MluI restriction sites. The N-terminal full-length mGFP PRR14 fusion was used in this paper as a base construct for mutagenesis. The mouse PRR14 expression vector was obtained from Origene Technologies, encoding a C-terminal fusion with TurboGFP (cat# MG209414). The mouse PRR14 ORF was transferred to the N-terminal mGFP vector from OriGene (pCMV6-AN-mGFP, cat# PS100048) using the Origene PrecisionShuttle system. For analyses of candidate modular LBDs from human, *Xenopus*, and gecko PRR14, and for expression of the isolated Tantalus domain, gene synthesis was used (Genewiz). LBDs and Tantalus sequences were synthesized with terminal SgfI and MluI restriction sites to facilitate cloning into the OriGene pCMV6-AN-mGFP vector using the PrecisionShuttle system. The candidate LBD modules were designed in most cases to include an SV40 NLS (PKKKRKV) at the N-terminal side to enhance nuclear import, with the final configuration being mGFP-NLS-LBD. The empty OriGene pCMV6-AN-mGFP vector was used as a GFP-only control, as needed. As a reference for the nuclear lamina, mCherry-LaminA-C-18 was used (Addgene plasmid # 55068, deposited by Michael Davidson; RRID:Addgene_55068). For FRAP experiments, pBABE-puro-GFP-wt-lamin A was used (Addgene plasmid # 17662, deposited by Tom Misteli; RRID:Addgene_17662) (Scaffidi and Misteli, 2008). For Tantalus–PP2A B56α pulldown experiments, an HA-tagged human B56α expression vector was used (Addgene plasmid # 14532, deposited by David Virshup; RRID:Addgene_14532) (Seeling et al., 1999).

### Site-directed mutagenesis

Site-directed mutagenesis was carried out using the Agilent Technologies QuikChange II XL Site-Directed Mutagenesis kit (cat# 200521). Mutagenic primers were designed using the Agilent web-based QuikChange Primer Design Program (www.agilent.com/genomics/qcpd). Mutagenic primers were purchased from Integrated DNA Technologies, Inc. (IDT). Mutagenic primer sequences are provided in the Table S3.

### Transfection

Plasmid transfections were performed using Lipofectamine 3000 (Invitrogen, cat# L3000008) according to the manufacturer's instructions. For some experiments, Lipofectamine 2000 was used (Invitrogen, cat# 11668030) according to the manufacturer's instructions. To create HeLa cells stably expressing mRFP–PRR14 or mCherry–Lamin A/C, HeLa cells were transfected with pCMV6-AN-mRFP-PRR14 or mCherry-LaminA-C-18, respectively using Lipofectamine 2000 according to the supplier's protocol, and then were selected using G418 (ThermoFisher, cat# 10131035) 48 h post-transfection. Next, cells were FACS sorted to enrich for mRFP/mCherry positive cells. For confocal imaging cells were plated on 8-well ibidi µ-slides (ibidi, cat# 80826) or glass-bottom 35 mm culture dishes (MatTek, cat# P35G-1.0-14-C), then transfected at 50% confluency and fixed 24 h post-transfection or imaged live. *Xenopus* S3 cells were transfected in 35 mm MatTek glass bottom dishes. S3 cells were transfected at 60–80% confluency using Fugene HD reagent (Roche, cat# E2311). Fugene HD (3 µl) was added to 400 µl Opti-MEM medium (GIBCO), along with 1 µg DNA, and the mix was incubated for 20 min. Next 400 µl Opti-MEM was added and the 800 µl volume was applied to the dish. Cells were cultured at 27°C and imaged 48 h post-transfection.

### Immunofluorescence

HeLa and C2C12 cells were fixed with 2% paraformaldehyde (PFA) (EMS, cat# 15710) for 10 min at room temperature, washed 3 times with DPBS (Gibco, cat#14190-136), then permeabilized with 0.25% Triton X-100 (ThermoFisher, cat# 28314) for 10 min. After permeabilization, cells were washed 3 times with DPBS for 5 min, then blocked in 1% BSA (Sigma, cat# A4503) in PBST [DPBS with 0.05% Tween 20, pH 7.4 (ThermoFisher, cat# 28320)] for 30–60 min at room temperature. Next, samples were incubated with primary antibodies for 1 h at room temperature, washed three times with PBST for 5 min, and then incubated with secondary antibodies for 30–60 min at room temperature followed by washing twice with PBST for 5 min. Samples were counterstained with DAPI solution (Sigma-Aldrich, cat# D9542) for 10 min at room temperature, then rinsed with PBS. Slides were mounted using 80% glycerol mounting media: 80% glycerol (Invitrogen, cat#15514-011), 0.1% sodium azide (Sigma-Aldrich, cat# S2002), 0.5% propyl gallate (Sigma-Aldrich, cat# 2370) and 20 mM Tris-HCl, pH 8.0 (Invitrogen, cat# 15568-025). Differential permeabilization was performed on HeLa cells transfected with the GFP–LBD constructs. Cells were fixed as described above at 24 h post-transfection and permeabilized with 0.25% Triton X-100 or 0.015% digitonin for 5 min at room temperature following the immunofluorescence protocol above, using anti-GFP and anti-Lamin A/C antibodies.

### Antibodies

The following antibodies were used in this study: anti-H3K9me3 (Abcam, cat# ab8898; 1:1000), anti-Lamin A/C (Santa Cruz, cat# sc-376248; 1:500), anti-Lamin B1 (Abcam, cat# ab16048; 1:1000), anti-GFP (Abcam, cat# ab290; 1:1000) and anti-HA (Santa Cruz, cat# sc-7392; 1:1000). The following Invitrogen secondary antibodies were used: donkey anti-rabbit (A10042), donkey anti-mouse (A10037), donkey anti-rabbit (A31573), and donkey anti-mouse (A31571).

### Image acquisition and analysis

Confocal immunofluorescence images were taken using a Leica TCS SP8 3X STED confocal microscope using 63×/1.40 oil objective. Images were acquired using HyD detectors in the standard mode with 100% gain. All images were taken with minimal laser power to avoid saturation. Confocal images were deconvolved using Huygens Professional software (Scientific Volume Imaging, The Netherlands). Some images in the Supplementary information were captured using Nikon TE2000 or Leica SP8 confocal microscopes. Confocal channel shift alignment was performed using 0.1 µm TetraSpeck fluorescent beads (Invitrogen, cat# T7279). Image analysis was performed using ImageJ software (National Institutes of Health, MD). Line signal intensity profile plots were created using the Plot Profile tool. Localization of the immunofluorescence signal at the nuclear periphery was determined using the signal at the nuclear periphery, measured using a mask of the nuclear lamina, expressed as a proportion of the total signal in the nucleus.

### Pulldown experiments

GFP-tagged PRR14 wild-type and mutant Tantalus fragments were used as bait proteins to measure interaction with wild-type and mutant HA-tagged PP2A B56α in transfected cells. ChromoTek GFP-Trap Magnetic Agarose affinity beads (GFP-Trap_MA, cat# gtma-20) were used for capture of GFP-fusion proteins. The GFP-Trap_MA is a GFP Nanobody/VHH coupled to magnetic agarose beads. HeLa cells (in 100 mm dishes) were co-transfected with pCMV6-AN-mGFP-PRR14 Tantalus constructs (or pCMV6-AN-mGFP) plus V245 pCEP-4HA B56α constructs using Lipofectamine 2000. Lysates were processed according to the protocol provided by Chromotek. Briefly, cells were scraped in ice-cold PBS and collected by centrifugation. After washing with cold PBS, cells were resuspended in 200 μl lysis buffer (10 mM Tris-HCl pH 7.5, 150 mM NaCl, 0.5 mM EDTA and 0.5% NP-40), plus protease inhibitors and 1 mM PMSF (Millipore Sigma cat# 10837091001). Tubes were kept on ice for 30 min, with pipetting, and centrifuged at 20,000× ***g*** for 10 min at 4°C. The supernatant was diluted to 500 μl with dilution buffer (10 mM Tris-HCl pH 7.5, 150 mM NaCl and 0.5 mM EDTA). An aliquot of beads was washed in the dilution buffer and finally resuspended in 500 μl of the same buffer. The diluted beads were added to the cleared lysate and were gently mixed for 1 h at 4°C. The beads were collected, washed with dilution buffer, resuspended in 100 μl 2× SDS sample buffer and heated for 10 min at 95°C to dissociate immunocomplexes. After removing the beads, samples were loaded on SDS–PAGE gels, and anti-GFP and anti-HA antibodies were used to detect GFP bait and HA-B56α prey proteins, respectively.

### FRAP

FRAP imaging was performed on a Leica SP8 laser scanning confocal system equipped with a heating chamber, using an HC PL APO 63×/1.4 NA CS2 oil immersion lens. HeLa cells were grown on 35 mm MatTek dishes. Cells were transfected with either N-terminal GFP-tagged human PRR14 or pBABE-puro-GFP-wt-lamin A. FRAP was performed at 16–20 h post-transfection, using 488 nm full laser power (65 mW). Five bleach iterations were performed (2–3 s total, in 0.5–0.8-s intervals). Recovery for GFP-PRR14 was measured for 58–60 s at 2-s intervals. Recovery for Lamin A was measured for 5.75–6.0 min at 5-s intervals. Data were plotted graphically using GraphPad Prism 8. EasyFrap software (Koulouras et al., 2018) was used to determine recovery half-time and R-square values.

### Statistical analysis

Statistical analyses were performed with Graphpad Prism 8.0.1 software (Graphpad Software, Inc.) using one-way non-parametric ANOVA (Kruskal–Wallis test) with Dunn's multiple comparison test, or unpaired non-parametric Student's *t*-test (Mann–Whitney test).

## Supplementary Material

Supplementary information
