# Supplementary material for: The PRR14 heterochromatin tether encodes modular domains that mediate and regulate nuclear lamina targeting
Source: J Cell Sci. 2020 May 27;133(10):jcs240416. doi: 10.1242/jcs.240416 (PMC7272351; doi:10.1242/jcs.240416)
Supplement: Supplementary information [file joces-133-240416-s1.pdf]

Supplementary information

Supplementary information includes seven supplementary figures, two supplementary tables, and two videos.

Table S1. Comparison of features of mammalian PRR14 and the PRR14L paralog with candidate PRR14 genes in non-mammalian species.

Click here to Download Table S1

Table S2. Summary of detection of PRR14 phosphopeptides in databases and our experimental work, BioID MS (mass spec).

Click here to Download Table S2

Table S3. Primers used in this study.

Click here to Download Table S3

Journal of Cell Science • Supplementary information

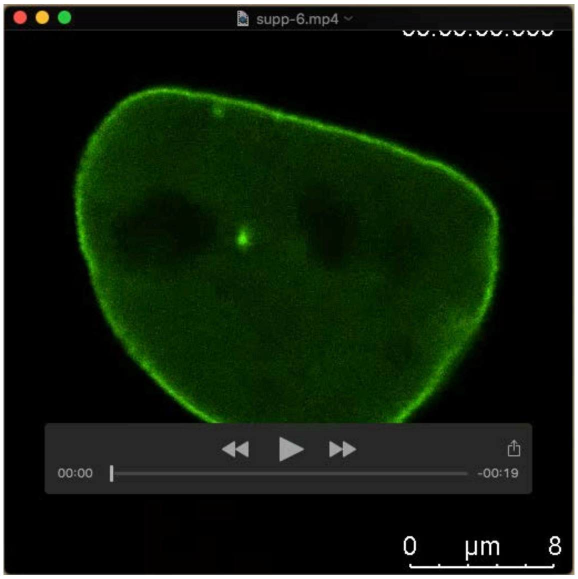

Movie 1. GFP-Lamin A fluorescence recovery at the nuclear periphery after photobleaching. Representative movie of a HeLa cell transfected with the GFP-Lamin A construct demonstrates slow Lamin A recovery at the nuclear periphery after photobleaching. Time stamp and scale bar are shown. Frames were captured in 5 s intervals.

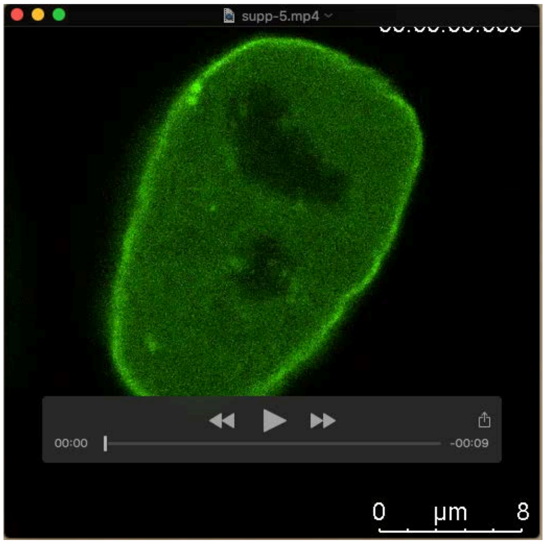

Movie 2. GFP-PRR14 fluorescence recovery at the nuclear periphery after photobleaching. Representative movie of a HeLa cell transfected with the GFP-PRR14 construct demonstrates rapid PRR14 recovery at the nuclear periphery after photobleaching. Time stamp and scale bar are shown. Frames were captured in 2 s intervals.

Journal of Cell Science • Supplementary information

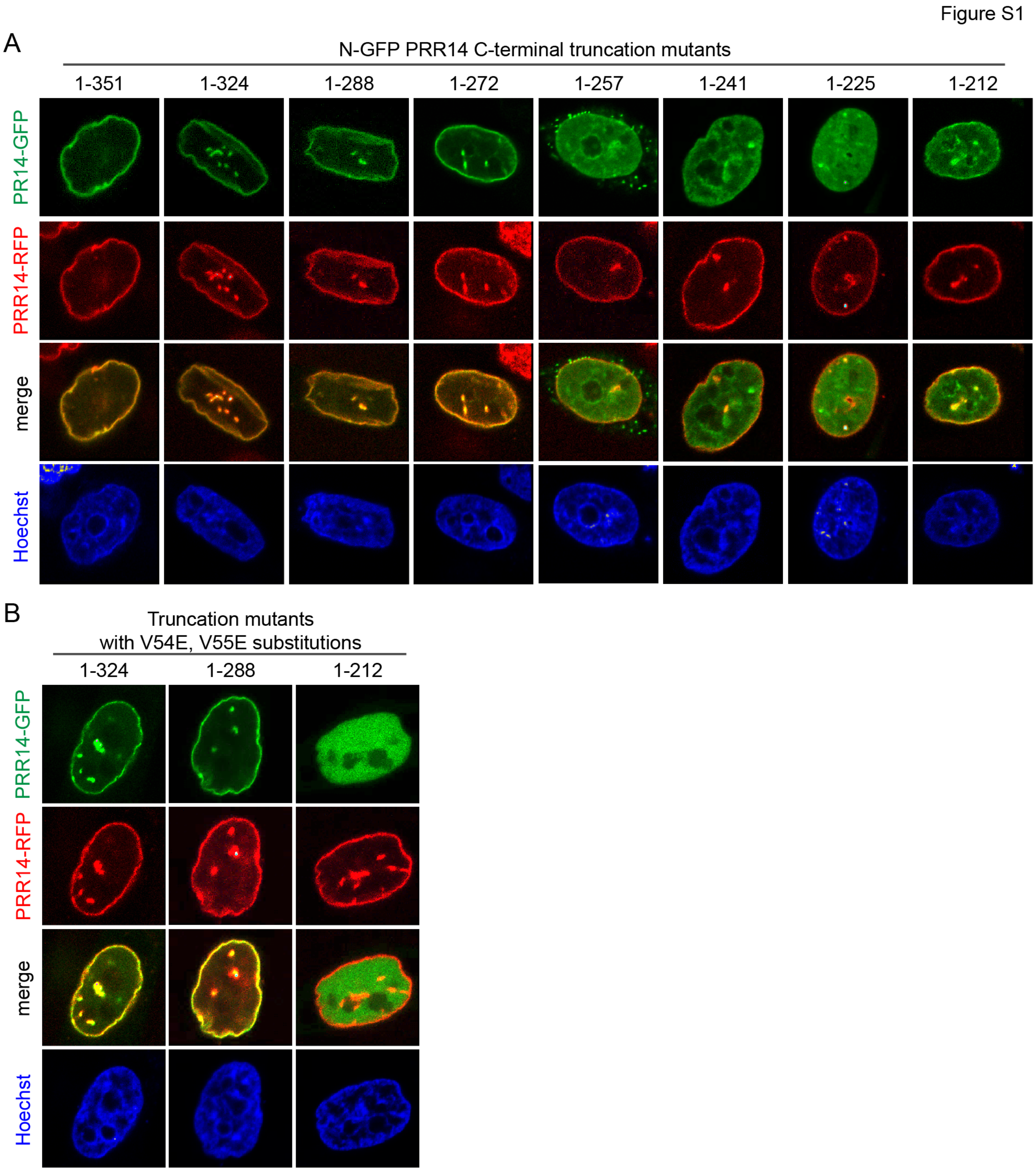

Figure S1. Deletion mapping of the PRR14 LBD. A series of PRR14 C-terminal truncations were created by introducing stop codons into the N-terminal GFP-tagged PRR14 reading frame. The end points of the C-terminal truncations are indicated. Representative confocal images of live HeLa cells stably expressing wild type mRFP-PRR14 transfected with **(A)** GFP-tagged truncation mutants and **(B)** composite mutants with substitutions in the LAVVL sequence (52-56) required for heterochromatin binding (V54E, V55E). Counterstained with Hoechst.

Journal of Cell Science • Supplementary information

Figure S2

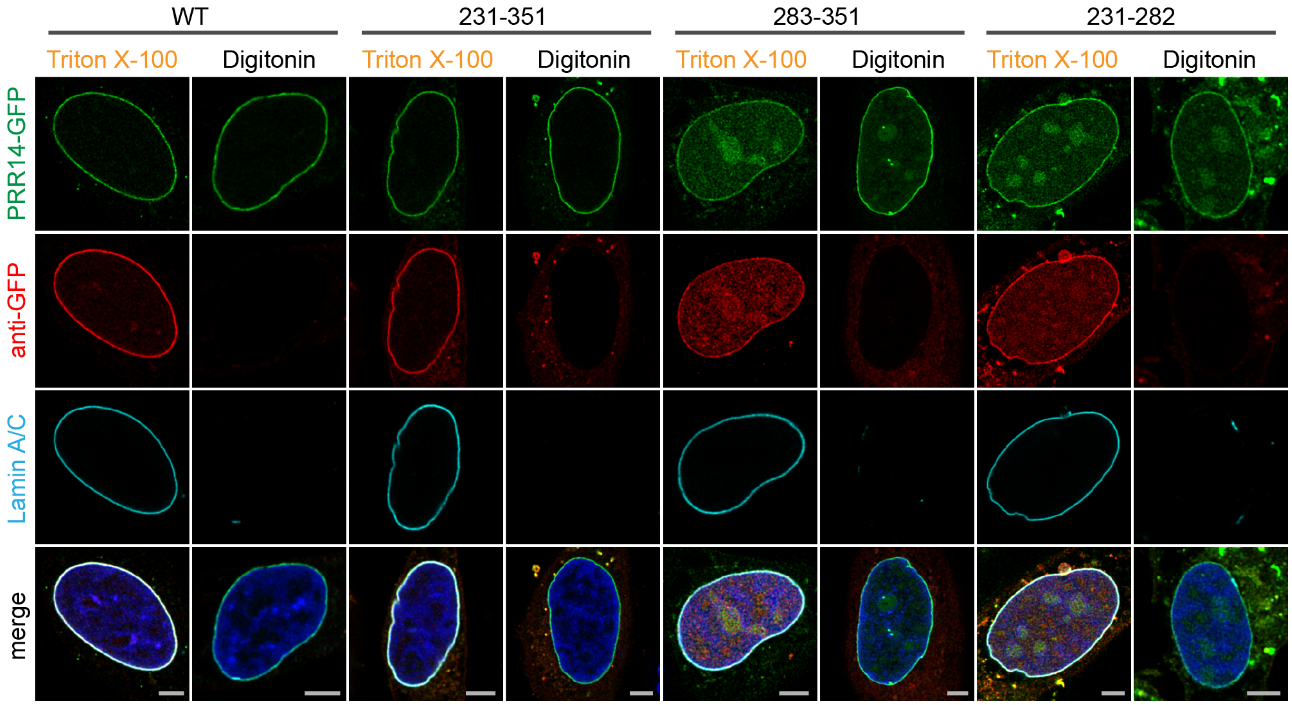

Figure S2. The PRR14 GFP-tagged 231-282 fragment localizes to the inner nuclear periphery. Representative confocal images of HeLa cells transfected with indicated GFP-PRR14 constructs and differentially permeabilized with Triton X-100 (plasma and nuclear membrane) or digitonin (plasma membrane only) to distinguish localization at the inner and outer nuclear periphery. Stained for GFP (red), Lamin A/C (cyan) and DAPI (blue). Scale bars: 5µm.

Journal of Cell Science • Supplementary information

Figure S3

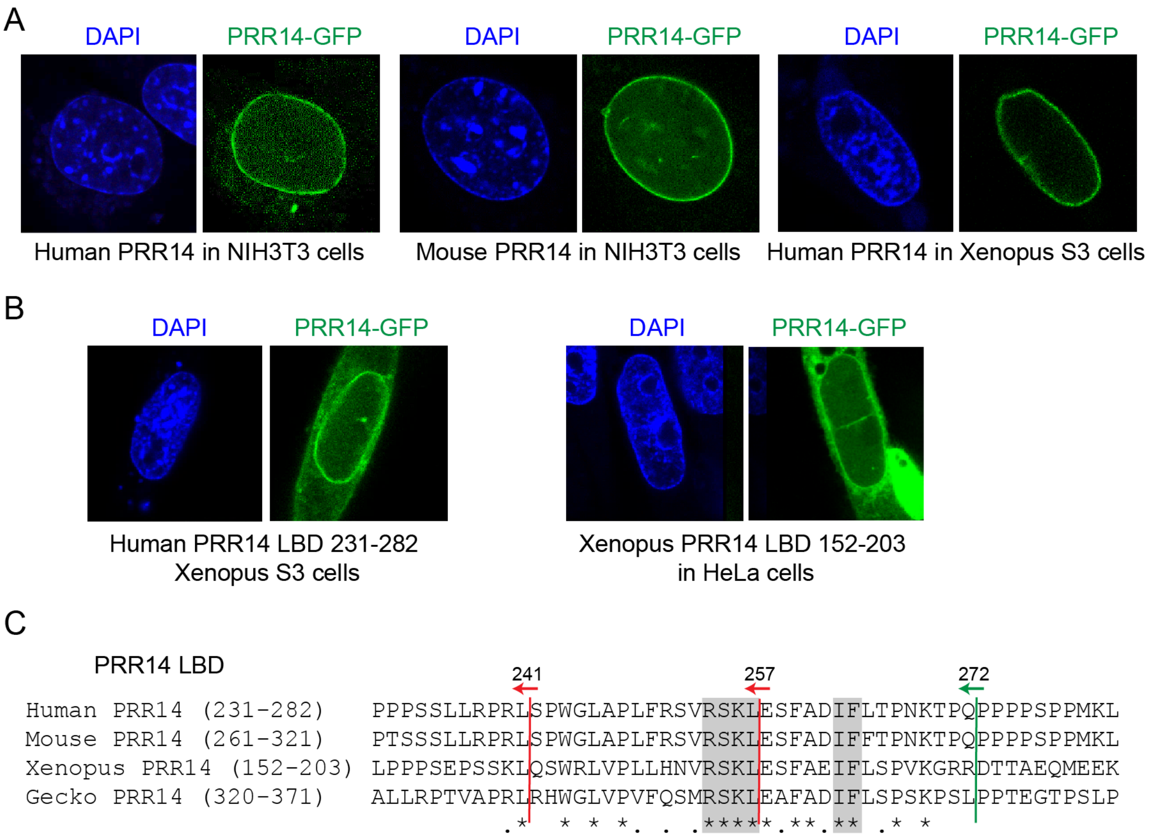

Figure S3. The mechanism for PRR14 nuclear lamina association is evolutionarily conserved. (A) GFP-tagged, full length human and mouse PRR14 proteins localize to the nuclear lamina in mouse cells. Human PRR14 localizes to the nuclear lamina in Xenopus cells. **(B)** GFP-tagged human PRR14 231-282 LBD localizes to the nuclear lamina in xenopus cells. GFP-tagged putative Xenopus PRR14 (see Table S1) 152-203 LBD localizes to the nuclear lamina in HeLa cells. **(C)** Manual alignment with no gaps of human and mouse, and putative xenopus and gecko LBDs. Amino acid identity (*) and similarity (.) are indicated. Conserved blocks chosen for analyses are indicated by shading. End points of relevant C-terminal deletions analyzed in Figure S1 are indicated. Green arrow indicates retention of nuclear lamina association and red arrows indicate loss of nuclear lamina localization. Deletion into the most conserved region of the human 231-282 LBD resulted in loss of nuclear lamina localization (Fig. S1).

Journal of Cell Science • Supplementary information

Figure S4

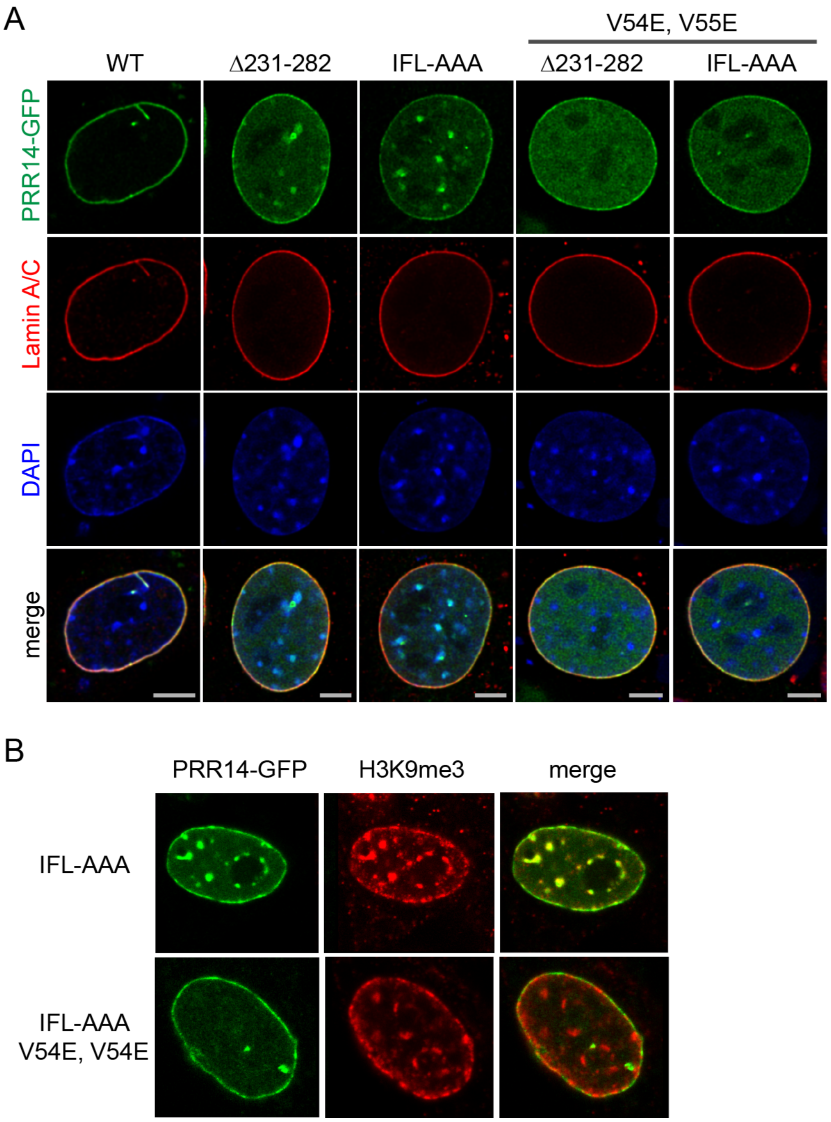

Figure S4. Composite mutations in the PRR14 231-282 LBD and HP1 binding site (V54E, V55E) reveal residual nuclear lamina association. (A) Representative confocal images of murine C2C12 cells transfected with the indicated PRR14-GFP constructs, similar to experiment shown in Figure 3B. The IFL-AAA and Δ231-282 constructs largely lost peripheral localization, and colocalized with chromocenters (heterochromatin). Composite LBD mutants also incapable of HP1/heterochromatin binding (V54E, V55E) lost chromocenter localization and spread throughout the nucleoplasm. These composite mutants showed some localization to the nuclear periphery, suggesting residual nuclear lamina binding. **(B)** Confocal images of HeLa cells transfected with the indicated IFL-AAA and composite IFL-AAA V54E, V55E mutants, with H3K9me3 staining. Complete colocalization of the IFL-AAA mutant with H3K9me3 chromocenters and peripheral H3K9me3 is observed, as expected. The composite IFL-AAA V54E, V55E mutant shows loss of chromocenter localization, also as expected. The bottom set of images were selected as depicting apparent high residual peripheral localization. H3K9me3 staining shows that localization of the composite mutant does not correlate with strong H3K9me3 heterochromatin signals at the periphery, indicating that the IFL-AAA (and Δ231-282) mutant likely retains residual nuclear lamina localization. Scale bars: 5μm.

Journal of Cell Science • Supplementary information

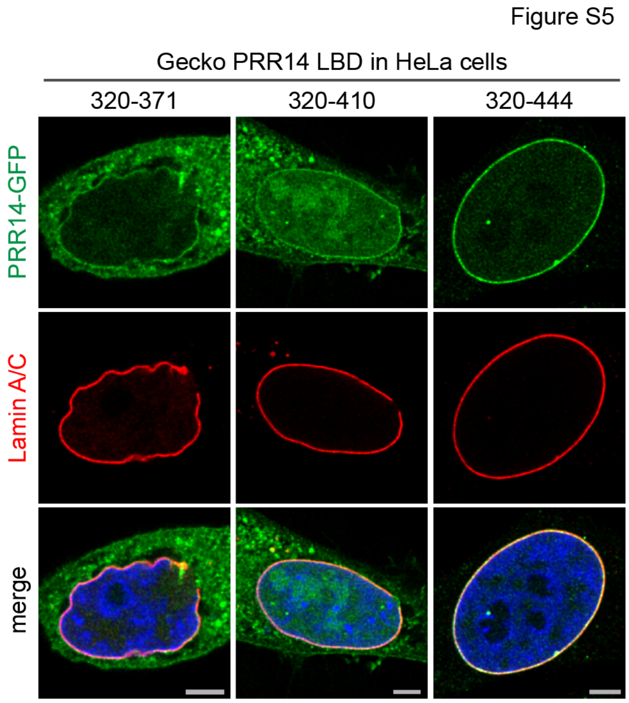

Figure S5. Mapping of the gecko PRR14 LBD. Representative confocal images of HeLa cells transfected with GFP-tagged gecko PRR14 320-371, 320-410, and 320-444 fragments (green), and stained with anti-Lamin A/C (red) and DAPI (blue). Scale bars: 5μm.

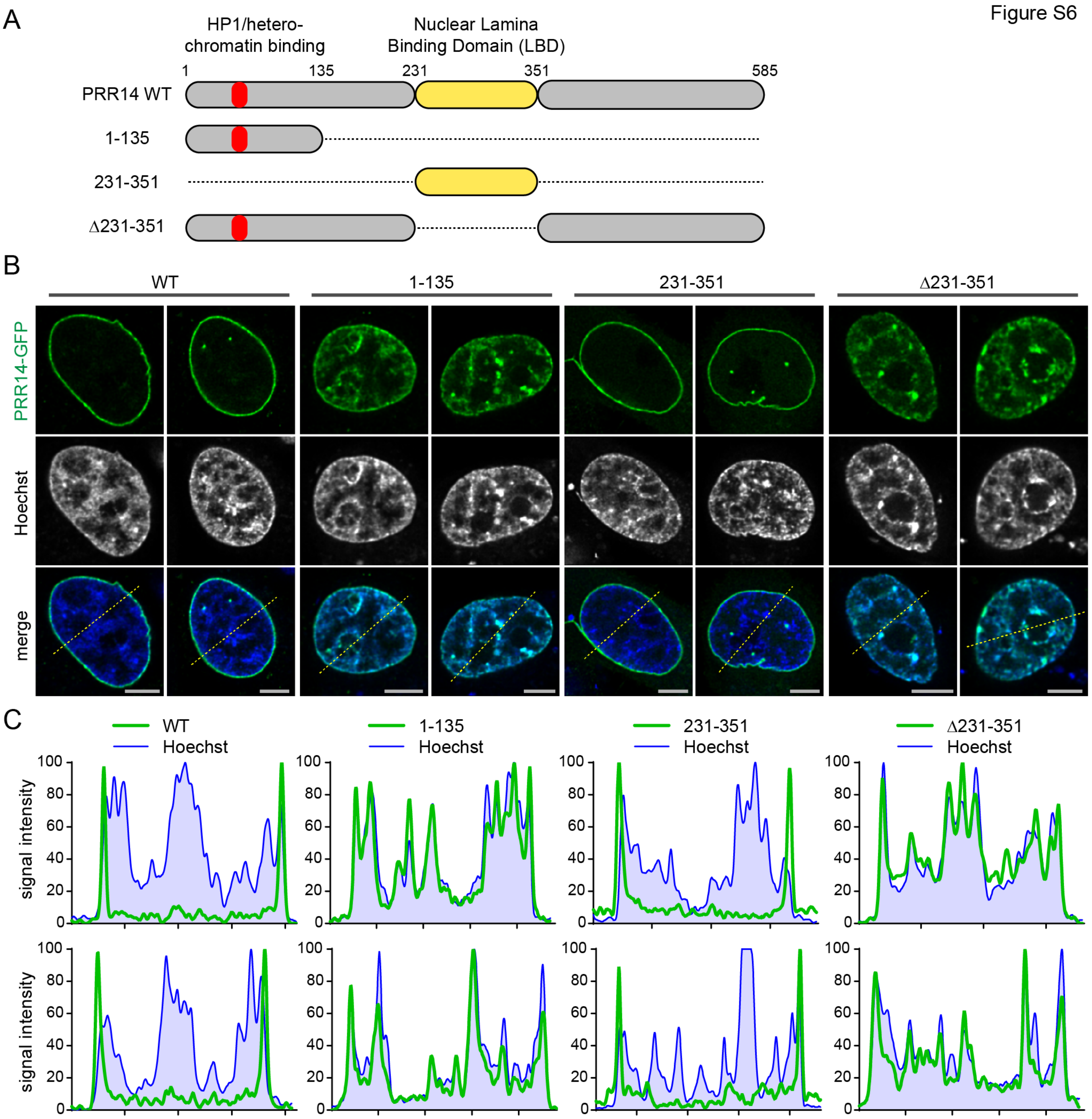

Figure S6. The 231-351 PRR14 LBD fragment does not localize with heterochromatin. (A) Schematic representation of PRR14 constructs used in Panels B and C to comprehensively map heterochromatin binding potential. The LAVVL HP1/heterochromatin-binding motif is indicated in red. **(B)** Two representative confocal images are shown of HeLa cells transfected with the indicated N-GFP-tagged PRR14 constructs, with Hoechst DNA staining (blue) **(C)** Line signal intensity profiles of corresponding images in panel B are indicated (see dashed lines in Panel B). Top and bottom graphs correspond to left and right images, respectively, for each construct shown in Panel B. In contrast to the PRR14 1-135 and Δ231-351, the 231-351 LBD fragment localized to the nuclear lamina and no colocalization with heterochromatin was observed. Scale bars: 5μm.

Journal of Cell Science • Supplementary information

Figure S7

```
● hPRR14    LTPMGLPRPIRLNKKEFSLEEIYTNKNYQSPTTRRTFETIFEEPRERNGTLIFTSSRK
● hPRR14L   LTPMGLPRPKRLKKKEFSLEEIYTNKNYKSPPANRCLETIFEEPKERNGTLISISQQK
  mPRR14    LTPMGLPRPIRLNKKEFTLEEIYTNKNYQSPTTRRTFETIFEEPRERNGTLIFTSSRK
  mPRR14L   LTPMGLPRPKRLKKKEFSLEEIYTNKNYKSPPASRCLETIFEEPKERNGTLISISQQK
● dTan                         EKIYTSRRMKK-FTPTNLETIFEEPSDENAA
                               * ***            *******   *
                              PP2A B56alpha-  [ LxxIxE
                           interacting motifs | F
● PP2A binding partners           (SLIM)      [ M
```

Figure S7. Alignment of Pfam Tantalus domains from mammalian PRR14, PRR14L and Drosophila Tantalus. Human and mouse PRR14 and PRR14L Tantalus domain sequences are shown, aligned to positions 145-174 of the 299 amino acid Drosophila Tantalus protein. The 145-174 Drosophila Tantalus region shown is only a subset of the 119-198 Drosophila Tantalus domain, as it was the only region showing significant homology. Human PRR14, PRR14L, and Drosophila Tantalus had been identified as PP2A interactors (see main text) implicating the conserved core sequences (shaded) in mediating PP2A binding. Subsequently, the L/F/MxxIxE SLiM motif was identified as a common recognition sequence for the B56alpha regulatory subunit of PP2A (see main text).

Journal of Cell Science • Supplementary information
